# Automated Patent Categorization and Guided Patent Search using IPC as Inspired by MeSH and PubMed

**DOI:** 10.1186/2041-1480-4-S1-S3

**Published:** 2013-04-15

**Authors:** Daniel Eisinger, George Tsatsaronis, Markus Bundschus, Ulrich Wieneke, Michael Schroeder

**Affiliations:** 1 TU Dresden, BIOTEC, Tatzberg 47/49, 01307 Dresden, Germany; 2 Roche Diagnostics GmbH, Nonnenwald 2, 82377 Penzberg, Germany

## Abstract

Document search on PubMed, the pre-eminent database for biomedical literature, relies on the annotation of its documents with relevant terms from the Medical Subject Headings ontology (MeSH) for improving recall through query expansion. Patent documents are another important information source, though they are considerably less accessible. One option to expand patent search beyond pure keywords is the inclusion of classification information: Since every patent is assigned at least one class code, it should be possible for these assignments to be automatically used in a similar way as the MeSH annotations in PubMed. In order to develop a system for this task, it is necessary to have a good understanding of the properties of both classification systems. This report describes our comparative analysis of MeSH and the main patent classification system, the International Patent Classification (IPC). We investigate the hierarchical structures as well as the properties of the terms/classes respectively, and we compare the assignment of IPC codes to patents with the annotation of PubMed documents with MeSH terms.

Our analysis shows a strong structural similarity of the hierarchies, but significant differences of terms and annotations. The low number of IPC class assignments and the lack of occurrences of class labels in patent texts imply that current patent search is severely limited. To overcome these limits, we evaluate a method for the automated assignment of additional classes to patent documents, and we propose a system for guided patent search based on the use of class co-occurrence information and external resources.

## Background

**Figure 1:**
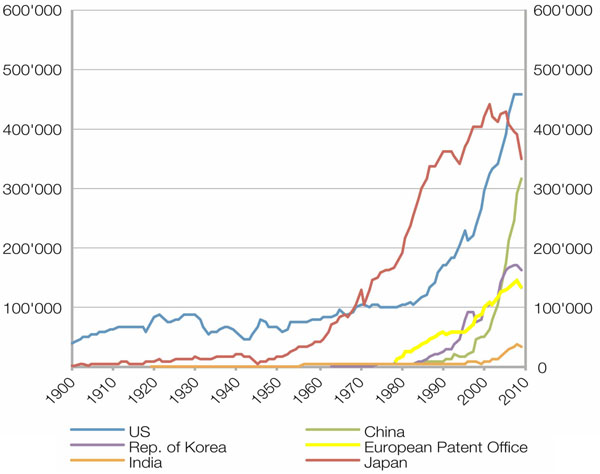
Number of patent applications per year for six top patent offices until 2010. With the exception of Japan, all offices are showing strong growth over the last two decades, (adapted from World Intellectual Property Report 2011)

As evidenced by a growing number of reports about various high-profile patent trials in recent years, having the necessary information about all relevant competitor patents can be vital to a company’s interests. At the same time, current research results are often first published in a patent and only afterwards in a journal. This makes patents also a potentially valuable source for academic research, although to our knowledge, most academic researchers are not using patents. The number of patent applications continues to rise, reaching an all-time high of almost 2 million worldwide in 2010 alone, with the number of granted patents in that year also setting a new record with more than 900,000 grants [1]. As can be seen in Figure 1 that was adapted from the World Intellectual Property Report 2011 [2] (published by the World Intellectual Property Organization (WIPO) [3]), five of the six top patent offices have experienced strong growth in the number of accepted patents over the last two decades, with Japan being the sole exception. While the figure shows small declines for all offices except China at the end of the last decade, these are believed to be consequences of the global financial crisis. Despite the continued effects of the crisis, the declines are expected to be temporary; this hypothesis is also supported by the record numbers mentioned above [1].

It is therefore important to have systems for patent search that are both comprehensive and accessible. The most natural way to search almost any document collection is by using keywords. Unfortunately, there are some obstacles to the sole use of keywords for searching patents. In particular, patent language is often extremely complicated. Additionally, many companies use very unspecific vocabulary in order to make the scope of their patents as broad as possible. At the very least, finding most or all relevant keywords will require a large time investment.

### International Patent Classification

As a consequence of the problems with keywords, professional searchers rely on the use of classification information [4,5]. Patents are classified into hierarchical systems of categories by patent offices. The International Patent Classification (IPC) [6] is the most common system - it is used by over 100 patent-issuing bodies worldwide [7] - and its hierarchy constitutes the base of other important systems such as the Japanese “File Index” [8], the German “Deutsche Feinklassifikation” (DEKLA) [9] and the new Cooperative Patent Classification (CPC) [10] that is now used by the United States Patent and Trademark Office (USPTO) as well as the European Patent Office (EPO). The IPC hierarchy is divided into eight sections that correspond to very general categories such as “Human necessities” (section A) or “Chemistry/Metallurgy” (section C). Each section is made up of numerous classes (e.g., A61), and each class contains multiple subclasses such as A61K. Each subclass is again divided into main groups (e.g., A61K 38/00), and for most main groups there are additional subgroups such as A61K 38/17. In order to improve readability, we will refer to all individual entries of the IPC as “classes” instead of the correct term for the respective level of the hierarchy.

As this example shows, individual entries of the hierarchy are mainly represented by alphanumeric codes. The corresponding definitions are complicated and often depend on each other. The following list represents the complete definition tree for the subgroup code from the example above.

• A

Human necessities

• A61

Medical or veterinary science; Hygiene

• A61K

Preparations for medical, dental or toilet purposes

• A61K 38/00

Medicinal preparations containing peptides

• A61K 38/16

Peptides having more than 20 amino acids; Gastrins; Somatostatins; Melanotropins; Derivatives thereof

• A61K 38/17

from animals; from humans

This example illustrates the necessity to also consider the superordinate code definitions in order to understand what kind of invention is represented by the given code. It also shows that the code alone does not accurately represent the hierarchy in all cases: While class 38/17 is directly subordinate to 38/16, there is no direct hierarchical connection between classes 38/16 and 38/15 (definition: “Depsipeptides; Derivatives thereof”). Finding the most relevant classification codes to be used for search therefore constitutes a significant challenge, especially for users with little experience.

As a consequence of the high complexity of searching patents, major pharma companies employ patent professionals to relieve their scientists of this difficult and time-consuming task. Their patent searches often combine keywords and classification codes (and possibly additional metadata) in a single query. Many researchers without access to such resources ignore patents in favor of more accessible scientific literature. However, as we mentioned above, they thereby risk missing a lot of current research results. This study intends to investigate ways for

1. assisting patent professionals in finding the relevant elements (keywords and classification codes) for their search queries more quickly and

2. enabling non-professionals to start using patents as an important additional information source.

In order to test the validity of using patent classification information for these tasks, we analyzed the code assignment in the patent domain and compared it with the assignment of Medical Subject Headings to documents in the biomedical literature database PubMed. Although PubMed has a considerably more narrow focus than the patents do, we consider this comparison a useful approach for the following reasons: First, PubMed represents (to our knowledge) the largest freely accessible collection of scientific documents (or more precisely, abstracts) indexed with a controlled vocabulary, making it a natural target for our comparison of document annotations. Second, as we will describe in the next subsection in more detail, the assigned terms are already used for improving PubMed searches, mirroring our plan for the IPC codes. And third, although our patent corpus contains patents from many different fields, we are mainly trying to improve patent search for the biomedical domain.

### Medical Subject Headings

The Medical Subject Headings (MeSH) are a controlled vocabulary thesaurus of biomedical terms curated by the National Library of Medicine (NLM). Similar to the IPC, the hierarchy starts with 16 very broad categories such as “Anatomy” or “Organisms” and gets much more specific in deeper hierarchical levels. The MeSH terms are used in the biomedical literature database PubMed as a document indexing system, i.e., for annotating documents with relevant terms that describe their content. PubMed users can therefore restrict their search to documents that have been annotated with some very specific terms in which they are interested. On top of that, MeSH terms are used to automatically improve the recall of PubMed searches through query expansion: By mapping keywords from a search query to MeSH terms, relevant documents are included in the search results even if they only contain synonyms or hyponyms of the original keyword. That means that even PubMed users who are completely unfamiliar with MeSH can benefit from the search improvements it makes possible.

The use of MeSH for PubMed searches is just one example for a controlled vocabulary that is assisting document search. Many text mining and document retrieval applications rely on text annotations with terms from existing taxonomies or ontologies - either by using existing manual annotations or by automatically assigning relevant terms. Systems based on this principle include GoPubMed [11] as well as EBIMed [12] and its sister application Whatizit [13]. All these systems are mainly or exclusively intended for use with Medline/PubMed documents; there is no comparable system for patents. Consequently, it is desirable to offer scientists an easier option to formulate patent queries that include classification information. In order to provide such assistance, it is important to have a clear understanding of the properties of both classification systems. In this paper, we therefore investigate differences between the IPC and the established MeSH hierarchy and their implications for patent search. As a solution to problems we discovered through our analysis, we propose two approaches: a system for the automated assignment of additional classes to patent documents and a guided patent search system that assists the user by offering query expansion suggestions derived from class co-occurrence data or using existing knowledge from external sources.

### Related work

The importance of MeSH for the biomedical field has led to extensive research: There are mature approaches for automatically assigning MeSH terms to documents [14,15], and MeSH terms are successfully used for query expansion [16]. MeSH has also been used in combination with patents, e.g., for tagging diseases [17].

IPC-related research is much more limited than for MeSH, but scientific interest has been growing over the last decade. Some publications explain the professional approach to patent search and classification information [18,19] while others identify problems and suggest solutions: Annies [20] points out that the limits of using keywords for chemical searches necessitate the inclusion of classification information, but warns that class assignments are incomplete. Parisi *et al. *[21] emphasize the same point even more, saying that existing assignments may be “subjective, incomplete or inconsistent and sometimes even random.” Despite these problems, Becks *et al. *[22] report drastic recall improvements for patent retrieval from the inclusion of classification information. Many publications cover methods for the use of existing assignment information for prior art search [23].

The automated assignment of classes to patents is an important issue for all patent offices. It is therefore not surprising that most of the initial research had direct connections to patent offices such as the European Patent Office (EPO) [24] and WIPO [25,26]. In later years, different workshops such as the Japanese NTCIR [27] and more recently the CLEF-IP evaluation track [28] added patent categorization tasks. The results from the published approaches vary depending on the hierarchical level that was used: Trappey *et al. *[29] report precision values slightly above 0.9 for a small subset of IPC subclasses and main groups, Tikk *et al. *[30] correctly identify up to 37% of main groups, and Verberne *et al. *[31] reach an *F*_1_-score of 0.7 for the subclass level in their best run. To our knowledge, there is only one prior effort to classify patents down to the lowest level of the IPC: Chen *et al. *[32] report 36% accuracy for that difficult task. To date, there is no in-depth analysis of either hierarchy and no systematic comparison of both hierarchies, although there are some papers dealing with MeSH [33,34] or IPC [5,35-37] in a more general way.

## Results and discussion

This section reports the results of our analysis of MeSH and IPC. In order to tackle problems we discovered in this comparison, we then discuss our efforts to automatically assign additional classes to patents and our investigation into possibilities for assisting patent searchers in utilising the classification for their search.

### Comparative analysis

Our analysis of MeSH and IPC can be divided into two parts: The first part concerns the respective hierarchies and terms of the systems themselves, while the second part examines their usage for document categorization. We analyzed the latter by collecting classification information from all patent applications to the European Patent Office (EPO) between 1982 and 2005 (over one million) as well as the annotations to all PubMed documents published by early 2011 (over 20 million). Our analysis has the goal of assisting patent search; we are therefore less interested in the reasons for any discrepancies than in their implications for search. Table 1 summarizes some core results of our analysis, and the following subsections give more detailed reports.

**Table 1:** Comparative analysis MeSH vs. IPC. The hierarchical structures are similar, but MeSH terms are shorter and more likely to occur in text. The number of MeSH annotations per document far surpasses the number of classes per patent.

Property	MeSH	IPC
number of hierarchy entries	54095	69487
number of unique entries	26581	69487
number of hierarchy levels	13	14

average string length main labels/class definitions	18	50
string length longest main label/class definition	104	596
string length shortest main label/class definition	2	3
average number of synonyms	8	0
occurrence of class labels in text	frequent	very rare

average number of annotations per document	9	2
number of unique annotations	25646	56599
proportion of documents with multiple annotations	86%	53%
proportion of documents with related annotations (same hierarchy tree)	81%	46%

#### Hierarchies and terms

**Figure 2:**
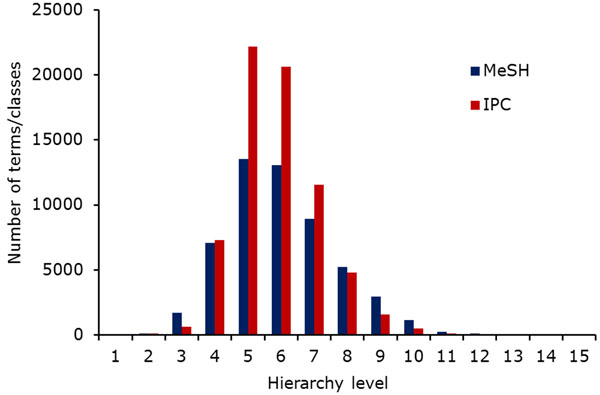
Terms/classes per hierarchy level. Both hierarchies expand in similar ways.

As Table 1 shows, the structural comparison of the hierarchies did not reveal any significant differences: Their sizes are in the same range (about 70000 IPC classes and 54000 entries in the MeSH tree), they have almost the same depth (14 levels for IPC, 13 for MeSH) and the node distributions are similar (cf. Figure 2). The main difference of the hierarchies is reflected in Table 1 in the distinction between the number of hierarchy entries and unique entries: While each IPC class can only be directly subordinate to exactly one other entry, MeSH allows its entries to have more than one father. This means that the same MeSH term can occur in multiple places inside the hierarchy. The terms on the other hand show two major differences:

• Emphasis on terms/concepts versus identifiers:

While MeSH assigns identifiers to its headings, the emphasis is clearly on the term itself. The IPC on the other hand is first and foremost a collection of alphanumeric codes which are signifying their place in the hierarchy. Unlike MeSH terms, these codes do not give an uninformed user any useful information about the patents that should be assigned to this class. This information is instead contained in additional class definitions that are more akin to MeSH’s scope notes. As an example, looking up the MeSH headings for a document about insects on PubMed will lead the user to the term “Insects”, not its identifier “D007313”. However, a patent about an immunoassay is assigned to class “G01N 33/53” (or one of its subclasses), and the definition of this class has to be checked separately if the user does not know it.

• Length of terms/occurrence in text:

As Table 1 shows, IPC definitions are usually much longer than MeSH terms. Most of them are also considerably more abstract and complicated, and many of them depend on their ancestor classes for a complete understanding, e.g., “from animal; from humans” (class A61K 38/17; cf. section *Background*). Unlike MeSH, the IPC also does not include any synonyms for the class definitions. All of these differences contribute to a very low probability of occurrence of class labels in text, while MeSH terms occur frequently [38,39]. In order to quantify how rarely IPC codes occur in text, we searched the complete texts of all 14600 documents from our test corpus *C*_7_3 (for a definition see *Training Corpora*) for their class definitions. Less than 2% of the documents contained their respective definition, and most of these hits were for class names that weren’t informative on their own (e.g., class C07K 14/47, “from mammals”).

As a consequence, IPC classes cannot be assigned to patents by simply extracting them from text. This is one of the main reasons for the much more extensive use of automated (pre-)annotation of PubMed documents compared to patents. One possible approach to solving this problem is the assignment of classes using machine learning methods, i.e., training a classifier on existing classification data to predict assignments for new data.

#### Usage for document classification

**Figure 3:**
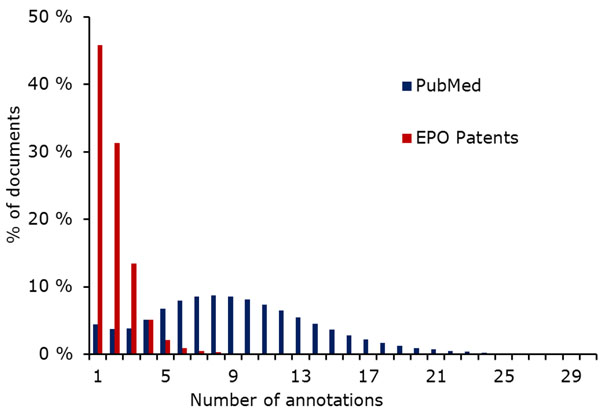
Percentage of documents with number of annotations. The average number of MeSH annotations per PubMed document is much higher than the number of IPC classes per patent.

**Figure 4:**
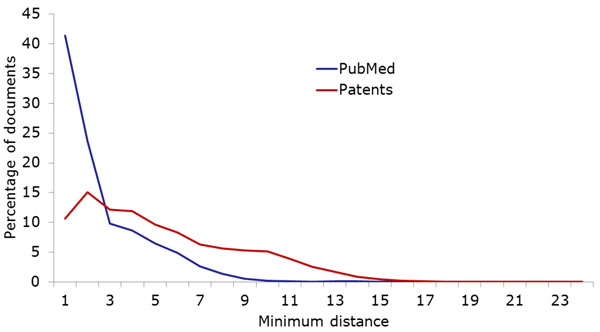
Minimum hierarchical distances of multiple annotations assigned to the same document. PubMed documents have more very closely related annotations than patents.

**Figure 5:**
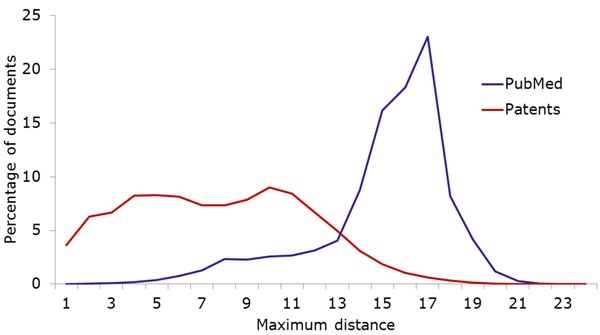
Maximum hierarchical distances of multiple annotations assigned to the same document. The maximum distance is considerably larger for PubMed documents than for patents, as is the difference between maximum and minimum distances.

IPC and MeSH are both used as classification/annotation systems for documents: all patent applications are assigned at least one IPC code, and all articles in participating journals are annotated with appropriate MeSH terms. As Figure 3 shows, the average number of MeSH annotations per document is much higher than the average number of IPC annotations per patent. Patents from our corpus had less than two assigned IPC classes on average, while PubMed documents have almost nine MeSH terms (cf. also Table 1). We also measured the diversity of IPC classes/MeSH terms assigned to the same document as follows: Given a hierarchy *H* (in our case either MeSH or IPC) and two entries *a* and *b* of the hierarchy, we define the distance between *a* and *b* as the length of the shortest path between them in *H.* For a subset *A* of *H* consisting of all annotations to a single document, we then define the maximum (minimum) annotation distance as the maximum (minimum) over the pairwise distances of elements of *A.* Since both MeSH and IPC are organized as a union of trees, we inserted one artificial root node into each hierarchy. For example, the most distant IPC classes assigned to one patent from our corpus have the definitions “Chemical Analysis” and “[Immunoassay] using isolate of tissue […]”. These classes are directly related, and the shortest path connecting them in the IPC hierarchy has length 3. On the other hand, many PubMed documents have annotations from different parts of the MeSH hierarchy: One example document is annotated with “Cyanides” from the “Chemicals and Drugs” tree as well as with “Risk Factors” from the “Health Care” tree. These terms show multiple aspects of the document, and their distance in MeSH is 14. As these examples show, the distance between terms hints at whether they belong to the same main tree of the hierarchy; below, we examine this property of the annotation terms more precisely. However, the distance also allows us to estimate the diversity of annotations from the same tree. Figures 4 and 5 show the minimum and maximum differences for PubMed as well as our patent corpus.

**Figure 6:**
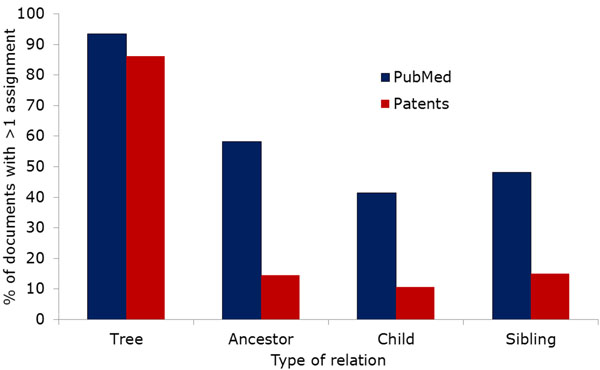
Hierarchical relationships of terms assigned to the same document. PubMed documents have considerably more closely related annotations, but the percentage of documents with annotations from the same main tree is almost equal for patents.

As Figure 4 shows, many PubMed documents are annotated with very similar MeSH headings, in many cases even pairing a term with its direct parent (i.e., annotations have distance 1). Arguably, this means that there is more redundancy contained in the MeSH annotations, since the parent term is implicitly assigned together with the child term. This situation is less common in our patent corpus, although there are also many patents that are annotated with closely related classes. The analysis of maximum distances (Figure 5) has the opposite result: While the maximum distances for patents do not differ too much from the minimum distances, the maximum distances for PubMed documents are much larger. The higher number of PubMed annotations is certain to play an important role in these differences. This result indicates that PubMed annotations cover a broader spectrum of aspects than the assigned patent classes. In addition to the path lengths between annotations, we examined the relations between annotations more directly by checking how often terms were co-assigned with closely related terms. Figure 6 shows the percentage of documents (among those with multiple annotations) that were assigned pairs of annotations with , it is much more common for MeSH annotations than for IPC codes to be co-assigned with sibling terms (i.e., terms that have the same parent term), their direct parent terms or more distant ancestors. On the other hand, it is very common for patents to get assigned to multiple classes from the same tree: Including patents with just one annotation, over 83% of all patents are classified into only one of the eight main sections of IPC. Since the main trees correspond to extremely general domains such as “Human necessities”, we believe that some aspects of many patents are not covered by the currently assigned classes.

#### Problems for IPC-based search

It could be argued that some of the described discrepancies are likely caused by differences in classification guidelines between patent offices and PubMed and may therefore be intended. However, that does not change the fact that both IPC and MeSH are used to improve search results on document corpora, and we believe that the use of IPC for that purpose comes with two serious disadvantages:

• Complexity of the classification system:

The complexity of the IPC terms causes significant problems for non-professional patent searchers. On the other hand, the exclusive use of keywords for searching patent databases often leads to bad results due to the complicated language used in patents.

• Sparse class assignments:

The low number of class assignments in combination with the relatively close relation of co-assigned classes indicates that relevant aspects of many patents are not covered by the existing class assignments. Consequently, the recall of patent searches using the classification may be lower than expected.

Given these disadvantages, patent search engines should offer additional functionality for helping the user find the required results. Since the class definitions are needed to understand the meaning of the class codes, the system must include easy access to them. Additionally, since many definitions depend on their ancestor classes, the engine should give the user an easy overview over the relevant parts of the hierarchy. Unfortunately, many popular existing engines such as Espacenet [40], Google Patents [41] and FreePatentsOnline [42] do not display this basic information on the same page as the patent.

### Assigning additional classes to patents

In the last section, we hypothesized that the low average number of class assignments to patents results in recall problems for patent search. The most straightforward way of dealing with this problem would be the assignment of additional classes, but due to the high number of patents as well as the high complexity of the classification system, this can only be done automatically.

As we described in the Related Works section, there have been multiple published approaches for the assignment of IPC codes to patents. These approaches are usually restricted to higher levels of the hierarchy such as the class or subclass level [29,31] or the main group level [29,30]. The WIPO has also made a patent categorization tool available, offering users the possibility to have documents categorized to any of these levelS [43]. To our knowledge, there is only one prior effort to classify patents down to the lowest level of the IPC [32]. While all previous approaches were solely focused on automatically recreating the existing assignments, it is our goal to find additional relevant classes that the patent was not originally assigned to. Consequently, we evaluate our method both for its ability to recreate assignments and the quality of additional class proposals.

Our system is based on the approach that was used by [15] for the automated assignment of MeSH terms to PubMed documents. After using the Maximum Entropy approach to build one binary classifier for each class, each learned model is applied to all documents that are supposed to be categorized. For all classifiers that put the document into the positive category with high confidence, the document is added to the corresponding class. As a result, we retrieve a set of classes for each document. We explain the method in detail in the Methods section.

#### Training corpora

In order to evaluate the results of our categorization efforts, we constructed training corpora from the EPO dataset that was also the basis of our previous analysis. We used three parameters to choose the sets of classes and documents that we used for these corpora:

• number of patents

Since the Maximum Entropy method improves with a growing number of training documents [15], it is reasonable to restrict the categorization task to classes that were used to annotate some minimum number of patents. We therefore excluded classes that were assigned to fewer patents.

• text length

While the EPO dataset contains the bibliographic data to all European patents, it doesn’t always include the complete texts. Since the classifiers rely on the text, we only used documents that surpassed a certain minimum text length.

• only primary classification/also secondary classification

Of the classes assigned to a patent, one is always emphasized as the primary one; i.e., the one that is supposed to correspond to the central aspect of the patent. When choosing training documents for a class, it might therefore be advisable to concentrate on patents with the primary classification.

We constructed one corpus with strict requirements (only widely used classes, long patents and primary classification) and another with more relaxed requirements (also less widely used classes, shorter patents and secondary classification). The details are presented in the *Methods* section. As a result of applying these requirements to our set of patents, the first corpus contains 73 classes while the second one is much larger with 1205 classes. In order to enhance the readability of this paper, we will refer to the first corpus as *C*_73_ and the second one as *C*_1205_ for the remaining sections of this paper. This size difference in connection with the expected higher quality of the documents due to the constraints we mentioned above should lead to better categorization results for *C*_73_ than for *C*_1205_.

#### Evaluation

With our initial evaluation, we tested our method’s ability to retrieve the classes that were actually assigned to the patents. Therefore, all of these classes were considered correct while everything else was considered wrong. Table 2 shows the macro-average scores (precision, recall and *F*_1_-measure) of all classifiers using 10-fold cross-validation for the confidence threshold 0.5; the use of other values is investigated below.

**Table 2:** Evaluation results for confidence threshold 0.5. The precision values are identical for both corpora, but recall is considerably higher for the smaller corpus.

Corpus	Precision	Recall	*F*_1_-measure
*C*_73_	0.88	0.90	0.89

*C*_1205_	0.88	0.84	0.86

As Table 2 shows, the results are for the most part encouraging, with most values approaching 0.9. The recall value is 6% higher for the smaller corpus. Applying t-test to the recall values from the common classes of both corpora (i.e., the 73 classes from *C*_73_) confirmed that this difference is statistically significant with extremely high confidence (*α* < 0.001). However, despite the size differences between both corpora, the precision values are equal. This may suggest that the *C*_73_ results are about as good as can be expected from the use of the Maximum-Entropy method on patent texts.

Table 3 shows the most influential word features for five models trained on IPC classes with biomedical relevance. While the positive features are in general very representative of the respective IPC class, most of the negative features seem to be useful mainly for excluding patents about information technology. We plan to investigate the influence that different methods for choosing negative documents (cf. *Methods* section) have on these feature sets.

**Table 3:** Most influential positive and negative classifier features. Features were extracted from five binary Maximum-Entropy classifiers trained on IPC classes with biomedical significance, leaving out stopwords and words with three or less characters. The occurrence of positive/negative feature words makes a document more/less likely to be assigned to the class.

IPC code	A61B 5/00	A61B 17/70	A61M 25/00	BO1L 3/00	G01N 33/543
class definition (abbrev.)	Measurement for diagnostic purposes	Spinal positioners	Catheter	Laboratory glassware	Immunoassay

	light	bone	catheter	sample	binding
	sensor	portion	distal	fluid	analyte
Pos. Features	blood	member	tube	channel	sample
	patient	screw	lumen	chamber	surface
	tissue	spinal	portion	surface	antibody

	layer	data	data	data	data
	acid	information	time	information	information
Neg. Features	sequence	signal	signal	image	network
	network	method	information	signal	channel
	cells	cell	control	recording	transmission

While precision and recall values around 0.9 are generally acceptable when a single classifier is used, these values are problematic in our situation - especially when applied to the corpus with many classes. It is important to note that there are two different learning tasks:

1. Given a class, find documents for this class.

2. Given a document, find classes for this document.

**Figure 7:**
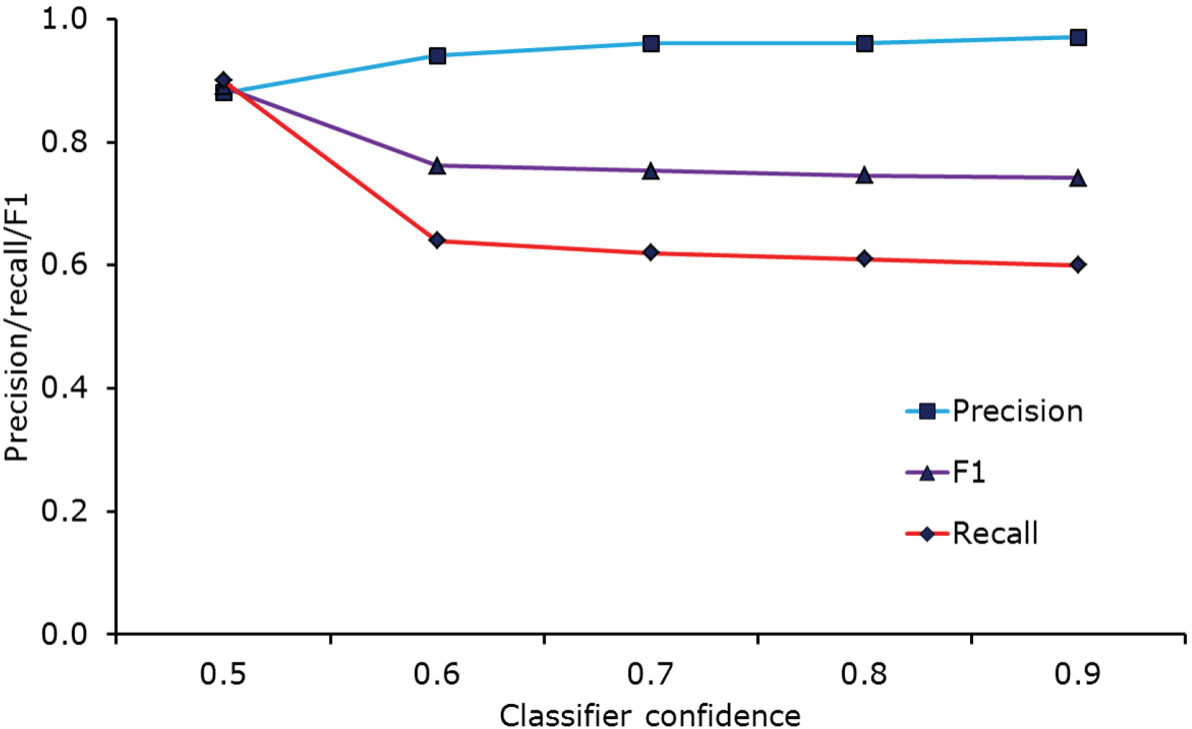
Classification results for corpus *C*_73_ depending on the confidence threshold. The *F*_1_ measure is highest for the value 0.5 due to a rapidly decreasing recall. Increasing the threshold further after the value 0.6 only leads to small changes.

For the first learning task, we have very promising results with precision, recall and *F*_1_-measure close to 0.9. The second task is more problematic however. Since we apply all classification models to all documents, most documents are assigned more than one hundred classes in the case of *C*_1205_. While this may appear to contradict our claim of the good performance of the individual classifiers, it does not: Even if every classifier makes the right decision in nine out of ten cases, applying more than 1000 such classifiers will still lead to many wrong decisions. This means that although the performance of the individual classifiers is satisfactory, we have to take additional steps in order to make its use for our intended application feasible. In order to reduce the number of class suggestions, we tried various higher values for the confidence threshold. In the PubMed/MeSH experiments detailed in [15], the highest *F*_1_-measure was reached for the confidence threshold 0.6. Unfortunately, our patent classifiers react less positively to raising the threshold, as can be seen in Figure 7: While raising the value from 0.5 to 0.6 clearly has a positive effect on precision, the corresponding drop in recall is much more severe and leads to a significantly lower *F*_1_-measure. Raising the value further only has negligible effects on the classification quality, leading to very slight precision increases and recall decreases.

**Figure 8:**
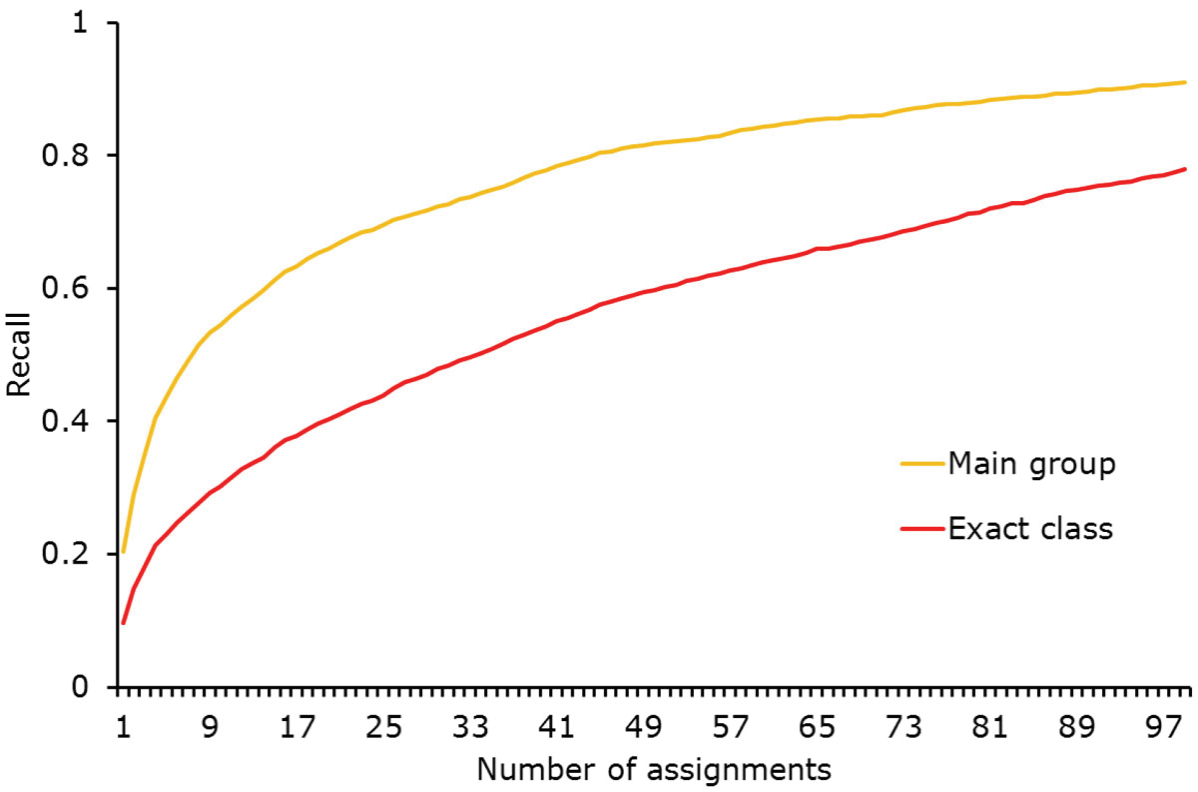
Recall for corpus *C*_1205_ depending on the number of assigned classes. The value grows rapidly until around ten classes, then continues growing at a slower pace.

Due to the high number of patent classes, our method’s precision would have to be very close to 1 in order to make the assignment of additional classes to all documents feasible. Unfortunately, since raising the confidence threshold only leads to moderate increases in precision, we cannot reach a value high enough for practical application of the method by itself. Still, since most queries also include a keyword component, it is possible to use the described approach to improve recall for such combined searches. Despite that, we also tried to filter the assignments of our approach in order to make it useful by itself: As before, we applied every classifier to every model. However, instead of setting a confidence threshold for the classification score, we decided in advance how many classes were supposed to be assigned to each document. After calculating all classification scores, we only retained the pre-determined number of highest-ranking classes. Figure 8 shows the recall of the method depending on the number of assigned classes, both for the exact class (i.e., the subgroup) and the more general main group. We calculated the main group recall by considering all subgroups below the closest main group as correct; in terms of our example from the *Background* section, for a patent from class A61K 38/17, also classes such as A61K 38/00 and A61K 38/16 are accepted. We chose to have the method assign ten classes in order to strike a balance between recall and precision. A small-scale manual evaluation of the results revealed that this method is able to recreate some assignments and to add relevant classes that were not assigned. As an example, patent *EP*1286824 about an “apparatus for clamping and releasing contact lens molds” was correctly assigned to class B29D 11/00 about the production of optical elements, and it was also assigned to the relevant class G02C 7/02 about lens systems - this class was not among the original assignments. However, even among the ten assigned classes for each patent, there were usually at least five completely irrelevant ones. The example patent about contact lens production was also assigned to class A61K 31/485 which is about medicinal preparations involving morphinan derivatives as well as class B29D 30/06 which is about pneumatic tyres or parts thereof. These results make the practical application of the method without any other filters doubtful. They are however not unexpected considering the slow precision growth that we pointed out in Figure 7: Our method effectively increases the confidence threshold further, reaching different values for each classifier. But since precision remains almost constant (albeit at a high level), this is still not enough to remove all irrelevant assignments.

However, we plan to investigate another possibility to filter out incorrect class assignments. As we will explain in *Proposing Additional Classes for Search*, we also did an analysis on pairs of classes that were frequently assigned to the same patent document. This data should also be useful for this task, since it is likely that classes that were never assigned to the same document do not cover similar subjects. An initial test had the result we were hoping for: For the example patent about contact lens production, our approach was able to filter out the incorrectly assigned classes about morphine and pneumatic tyres, since they had never been assigned to the same patent as class B29D 11/00 that had been assigned by the patent office. On the other hand, the newly assigned relevant class about lens systems did co-occur with class B29D 11/00 and was therefore not filtered out.

### Guided patent search

An additional possible way for tackling the problem of low class assignment is the expansion of user queries to make up for the “missing” assignments. Since professional patent search queries are a combination of class codes and keywords in most cases, we investigate ways to expand both of these components in the following subsections.

#### Proposing additional classes for search

If a user query contains a class code, it can be assumed that the user is confident of the relevance of that class. In order to find closely related classes to suggest to the user, we analyzed the class co-assignments in our patent corpus. We collected all pairs of classes that were assigned to the same patent and ranked them both on the absolute number of co-assignments and the relative number in the form of their Jaccard-Index. We hypothesize that pairs of classes with high ranks in either ranking are related closely enough that many searches for one of the classes will also have additional relevant results in the second class. We therefore propose to suggest these frequently co-occurring classes to the user for query expansion. In order to ensure the quality of our suggestions, we based the co-assignment statistics solely on the existing EPO assignments. However, we plan to investigate the effect of including classes that were added by our automated method.

**Figure 9:**
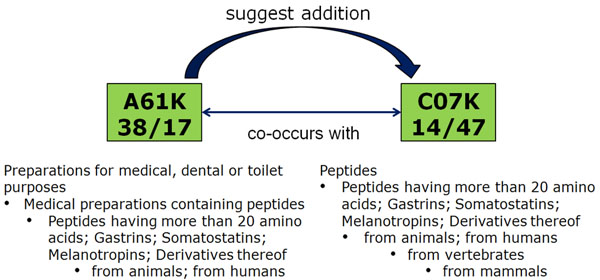
Example for semantically related IPC classes without any hierarchical relation, detected using co-assignment information.

**Figure 10:**
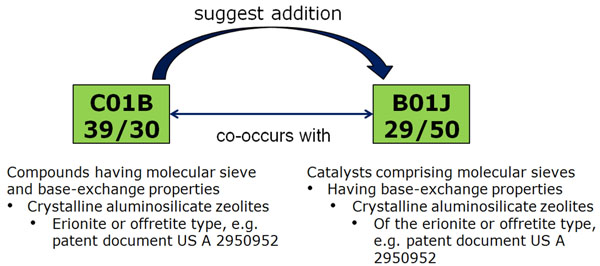
Second example for semantically related IPC classes without any hierarchical relation, detected using co-assignment information.

Many resulting class suggestions are from the same hierarchical tree but not directly related, i.e., they cover patents with very similar aspects to the ones searched for by the user. Additionally, the rankings include pairs of classes from completely separate parts of the hierarchy that are also highly related; in many cases, they can be considered to represent different points of view. Figure 9 shows one example of such a pair of classes, including their definition hierarchy. The left class is clearly more application-oriented than the right one, since it deals with “medical preparations containing peptides” while the right class concentrates on the peptides themselves. However, we argue that many searchers interested in patents from one class will also find relevant patents in the other one. We used the professional patent search tool Thomson Innovation to find out how recall is affected when only one class is used for search. For these example classes, searching for only the first class leads to over 50% missed possible results, and searching only for the second still leads to 25% missed results. The situation is similar for the pair of classes shown in Figure 10, also detected using co-assignment information.

**Figure 11:**
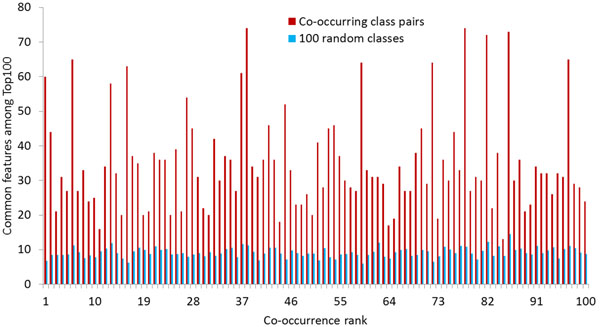
Classifier feature overlap among the Top 100 features for frequently co-occurring and random classes. The overlap is generally much higher for co-occurring classes, showing the significance of co-occurrence information.

In order to give a more general evaluation of how meaningful the class co-occurrences are, we used our categorization results. Since we trained binary classifiers for a large number of classes, we can compare their feature sets to each other. We chose the 100 most frequently co-occurring pairs of classes as well as 100 additional random classes. We then calculated the number of common features between different classes among the 100 top features for each classifier. Figure 11 shows these numbers for the co-occurring pairs compared to the average for all 100 random classes. For all co-occurring class pairs, the overlap is considerably higher than for the randomly chosen classes. On average, the co-occurring classes have 35 common features, while the random classes only have nine. This shows that the co-occurrence information is useful for finding related classes.

#### Proposing additional keywords for patent searches

As we showed above, it is possible to find additional relevant classes to expand user queries based on class co-occurrence. Another option for query expansion would be the suggestion of additional keywords to the user. The following subsections describe our efforts to expand patent queries with additional keywords extracted from various sources.

##### 1. Extracting keywords from classes

The most straightforward way of turning class codes into keyword suggestions is by considering the corresponding IPC definitions - both by extracting keywords directly and exploiting the morphosyntactic structure of definitions where possible. For definitions containing lists of related terms, we use the system described in (Fabian et al., 2012) to find additional terms with the same relation and suggest the top-ranking ones to the user. As an example, the suggestions for the IPC definition “Orthopaedic devices […] such as splints, casts or braces” include the relevant terms “slings”, “collars”, and “crutches”. For a baseline Boolean keyword query simply connecting the terms with “OR”, the result set almost doubles in size after the inclusion of the generated sibling terms. Our system detected 3053 IPC classes (≈ 4%) that contain enumerations and can therefore in principle be used in this way for query expansion. Taking this approach one step further, established NLP techniques can be used to extract keywords from the patent texts that belong to the query classes. In a way, this is also what we did for our categorization efforts: Each classifier gives weight parameters to the words contained in patent documents, with high values corresponding to words that are typical for the class. Table 3 shows the word features with the highest values for five IPC classes with biomedical relevance, demonstrating that this approach is able to discover useful search terms.

##### 2. Extracting keywords from external ontologies

Existing ontologies are another possible source for additional keywords. If an ontology term can be matched to an IPC class definition, any additional information contained in the ontology about the term (e.g., its synonyms) can be used to add suggestions for the user. As a proof of concept, we used the annotation pipeline from [11] to map MeSH terms to an IPC subset with biomedical relevance. For that purpose, we selected all subclasses of the IPC class “A61K” with the definition “Preparation for medical, dental or toilet purposes” (981 subclasses). The annotation results provided at least one MeSH term for 865 of these classes (88%), and three or more terms for 466 classes (48%). Many IPC classes were matched with very relevant MeSH terms, e.g., class A61K 48/00 (“medicinal preparations containing genetic material which is inserted into cells of the living body to treat genetic diseases; gene therapy”) with MeSH terms including “Genes”, “Cells” and “Gene Therapy”. On the other hand, there were also incorrect matches, often due to shortened MeSH synonyms. For the example class, the MeSH term “Containment of Biohazards” was considered a match because the word “containing” in the class definition was mapped to “Containment” which MeSH lists as a synonym. Since our system proposes expansion terms to the user instead of automatically adding them, this high level of coverage represents a valuable addition despite the inclusion of some false positive annotations.

The availability of a domain ontology also makes enhanced sibling generation possible: If an IPC definition contains a MeSH term as well as one of its child terms in the form of an example, it is reasonable to assume that all other child terms are also relevant. Following this intuition, IPC definitions of this form (e.g., “Sulfonylureas, e.g. glibenclamide, tolbutamide, chlorpropamide”) lead to term suggestions with very high precision (for the example: “Carbutamide”, “Acetohexamide”, etc.). Of the biomedical IPC subset, this was possible for 72 classes (7%).

##### 3. Repurposing class-keyword mappings for class suggestion

After keywords have been mapped to IPC classes in the proposed ways, the mapping data can also be used in the opposite direction: If the user enters a keyword that has been mapped to an IPC class, this class can be suggested to the user for expanding his query. If the class definition is displayed with the suggested class code, even users unfamiliar with the IPC can profit from classification information. This is especially true for the biomedical domain, since the availability of detailed domain ontologies leads to very precise class suggestions. The same approach applies again for the keywords that were selected by our classifiers ass representative of their class. The WIPO website used to offer similar functionality but it was not made clear what the system’s class proposals were based on and the service was stopped in November of 2012 without further explanation.

## Conclusions

We investigated possibilities for giving patent searchers access to the same advantages that are offered for PubMed through MeSH annotations. Our analysis of MeSH and IPC showed some unique characteristics of the patent domain, most importantly complex class definitions that rarely occur in text as well as a low number of class assignments. These discrepancies must be considered during the development of a patent retrieval system. We proposed ways to overcome these problems by combining two complementary approaches: the assignment of additional patent classes as well as the development of specialized components for a guided patent search system. Our experiments showed that automated patent categorization using the Maximum Entropy approach offers promising results with *F*_1_-measure values above 0.85 for individual classifiers. Including these newly assigned classes in a patent retrieval sytem by combining them with search keywords can offer considerable improvements to patent search. Additionally, we demonstrated that class co-occurrence data can provide valuable information to users and that existing ontologies and taxonomies such as MeSH can benefit the patent searcher by taking existing domain knowledge into account.

## Methods

This section describes in more detail the methods we used for our experiments. The first subsection explains our analyses of MeSH and IPC, and the following subsection concerns our experiments with patent categorization.

### Analysis of MeSH and IPC

Both analyses were carried out in two steps: We first retrieved and analyzed the terms and their hierarchical relationships, and then the annotations to our document corpora.

#### MeSH

For our analysis of the MeSH hierarchy, we used the XML version of MeSH 2012 retrieved from the MeSH homepage [44]. We extracted all MeSH terms with their MeSH IDs as well as the tree numbers from the file. The tree numbers were then used for reconstructing the hierarchy. We implemented graph-based methods for calculating different hierarchical properties such as the number of nodes per hierarchy level. For the PubMed/MeSH annotation analysis, we used the complete Medline dataset with MeSH annotations, downloaded from PubMed on September 22, 2011. After extracting the necessary information about documents and annotations, we analyzed it using a custom implementation, calculating different characteristics of the data such as the average number of annotations per document.

#### IPC

We reconstructed the IPC hierarchy using HTML files available from the WIPO homepage [45]. After manually entering the eight sections of the IPC with their definitions as top nodes of the hierarchy, our implementation automatically extended the hierarchy step by step: Each section file (e.g., “A.htm”) contained all main classes of the section, allowing us both to add them to our representation of the hierarchy and to retrieve the corresponding HTML files. Then the subclasses were extracted from the main class files (e.g., “A01.htm”), and the main groups and subgroups from the subclass files (e.g., “A01B.htm”). Since the class codes do not correctly reflect the father/child relationship between entries at the subgroup level (cf. section *Background*), we used the dot representation in the files to ensure the accuracy of our representation of the hierarchy. Class definitions were also extracted from the files in string form; images contained in a number of chemistry-related class definitions as well as references to related classes were removed. The analysis of the hierarchy was carried out using a slightly modified version of our MeSH implementation, leading to directly comparable results.

For the patent annotation analysis, we used XML files published by the EPO via their subscription-based “Product 14.12” [46]. We used the complete set of patent applications from the years 1981 to 2005, and we extracted document numbers as well as all classification information from the files. The reason for our exclusion of more recent patents was a change in EPO publication policies and data formats. Since there have been multiple updates to the IPC that are not reflected in the EPO’s files, we decided to use the 2006 version of IPC in order to minimize the number of class assignments that could not be matched. The document numbers were used to make sure that different versions of the same patent were not counted multiple times. For the classification information, we extracted both primary and secondary classification codes and combined them into one set of codes per patent. We again used a modification of the PubMed implementation to perform our analyses of the data.

### Automated patent categorization

As we described in section *Results and Discussion*, our approach to assigning additional classes to patents was based on [15], where a Maximum Entropy (MaxEnt) approach was used for document annotation with MeSH terms: For each MeSH term that was to be used for document annotation, a binary MaxEnt model was learned from already annotated documents and applied to new ones. We applied the same principle to patent classification, learning IPC classifiers from existing patent documents with classes manually assigned by professional patent examiners. The following subsections describe the Maximum Entropy approach in general as well as the details of our implementation.

#### Maximum Entropy

The Maximum Entropy approach estimates a probability distribution from existing data, based on the assumption that the distribution should be “as uniform as possible” if no external knowledge is available. This principle also gave the approach its name: *Entropy* measures the uncertainty of the outcome of a random variable, and its value is *maximized* if the random variable is uniformly distributed. Intuitively, this can be seen through the example of a coin toss: Its uncertainty is largest for a fair coin; if the coin is known to have a higher probability to show heads, it is easier to guess the next toss. Maximum Entropy has been used for various tasks in Natural Language Processing (e.g., language modelling [47] and part-of-speech tagging [48]) since the mid-nineties and was first proposed for text classification in 1999 by [49].

For this purpose, the existing data are documents that have been labeled with certain categories (the *training set*), and the probability distribution that is estimated by the approach is used to assign classes to new documents (the *test set*)*.* In order to do that, features are extracted from the training set. A feature is a measurable property of the documents, e.g., the number of occurrences of a certain word in the text or the year in which the document was published. For estimating the probability distribution, each feature *f_i_* is assigned a parameter *λ_i_* with initial value 0. Based on the relationship between feature values and class assignments in the training documents, these parameters are then updated iteratively until they converge. The result is a probability distribution based on the chosen features weighted by the corresponding parameters. In order to assign classes to a new document, it is then only necessary to input the document’s feature values into the distribution to get a classification score.

MaxEnt can be used for binary classification (i.e., one of two classes is assigned) as well as multi-class classification (one of multiple classes). Since our goal is multi-label classification (i.e., a subset of all classes should be assigned), we trained one binary classifier for each class and applied all classifiers to each document.

#### Implementation

Our corpus was again a subset of the EPO dataset we used for the IPC analysis. For the classification, we used patents published after 2005 and before July 2012. As we described in section *Results and Discussion*, we constructed two corpora by applying three different criteria: the number of patents, the text length and the optional restriction to primary classification. We included all classes that had the required number of patents fulfilling the text length requirement. In the first corpus, only patents that had the class as primary classification were counted. We then collected the required number of patents for each class by randomly choosing from the complete set. Table 4 shows the values we chose for the parameters as well as the number of classes that fulfilled the requirements and the resulting number of patents per corpus.

**Table 4:** Training corpora for patent categorization. *C*_73_ has more patents per class with longer text and only primary classification.

training corpus	number of patents/class	minimum text length	restricted to primary classification	number of classes	total number of patents
*C*_73_	200	8000 characters	yes	73	14600

*C*_1205_	100	2000 characters	no	1205	120500

We used the Java API of the open-source machine-learning toolkit Mallet (version 2.0.7) [50] for our classification efforts. The pre-processing was done in two steps: For each of the patent documents that were chosen from the EPO corpus for inclusion in *C*_73_ or *C*_1205_, we first created a text file that contained all text fields from the corresponding XML file. We then created a feature vector from each text file by using both existing and custom implementations of Mallet’s *Pipe* interface. The classifiers were trained by executing the *train* method from the Mallet class *MaxEntTrainer.*

The training sets for each classifier were constructed as follows: For the positive set, all patents that the corpus contained for the respective class were included. For the negative set, a few different approaches were investigated by [15]. Since the differences were very small, we decided to use the most simple option: We randomly chose the same total number of patents as in the positive set from the set of all other classes. In order to avoid the over-representation of individual classes, we shuffled all these classes and randomly selected one document from each of them in turn. Despite taking that step, the negative features seem to be overly concentrated on separating very distant technological fields and less useful for detecting subtle differences between classes (cf. Table 3). We plan to investigate different possibilities for constructing the negative set, e.g., increasing the number of documents from fairly similar classes. However, while this may help fine-tune the negative features, it is possible that the currently high quality of the positive features will suffer.

We used 10-fold cross-validation and calculated the macro-average scores (cf. Table 2). Since the cross-validation methods that are included in Mallet do not conserve the ratio of positive and negative training documents, we implemented a custom method for this task as well as for the evaluation of the categorization results.

Since both our approach and our objective for patent categorization differ considerably from the previous approaches we mentioned in the *Background* section, comparing the results directly is not possible: Almost all existing approaches are restricted to higher levels of the hierarchy, and all of them are used for assigning one single class instead of sets of classes.

## Competing Interests

The authors state that they have no competing interests.

## Author’s contributions

DE, MS conceived the ideas, analysed the data, and wrote the manuscript. DE implemented code. MB, UW, GT contributed to discussions.

## Declarations

This work was supported by Roche Diagnostics GmbH and the DFG projects GeneCloud and Hybris.

This article has been published as part of *Journal of Biomedical Semantics* Volume 4 Supplement 1, 2013: Proceedings of the Bio-Ontologies Special Interest Group 2012. The full contents of the supplement are available online at http://www.jbiomedsem.com/supplements/4/S1
